# Identifying genetic factors that contribute to the increased risk of congenital heart defects in infants with Down syndrome

**DOI:** 10.1038/s41598-020-74650-4

**Published:** 2020-10-22

**Authors:** Cristina E. Trevino, Aaron M. Holleman, Holly Corbitt, Cheryl L. Maslen, Tracie C. Rosser, David J. Cutler, H. Richard Johnston, Benjamin L. Rambo-Martin, Jai Oberoi, Kenneth J. Dooley, George T. Capone, Roger H. Reeves, Heather J. Cordell, Bernard D. Keavney, A.J. Agopian, Elizabeth Goldmuntz, Peter J. Gruber, James E. O’Brien, Douglas C. Bittel, Lalita Wadhwa, Clifford L. Cua, Ivan P. Moskowitz, Jennifer G. Mulle, Michael P. Epstein, Stephanie L. Sherman, Michael E. Zwick

**Affiliations:** 1 Department of Human Genetics, Emory University School of Medicine, 300 Whitehead Biomedical Research Building, 615 Michael St., Atlanta, GA 30322 USA; 2 Department of Epidemiology, Rollins School of Public Health, Emory University, Atlanta, GA USA; 3 Division of Cardiovascular Medicine and the Heart Research Center, Oregon Health and Science University, Portland, OR USA; 4 Sibley Heart Center Cardiology, Department of Pediatrics, Children’s Healthcare of Atlanta, Emory University, Atlanta, GA USA; 5 Kennedy Krieger Institute, Baltimore, MD USA; 6 Department of Physiology and the Institute for Genetic Medicine, Johns Hopkins University School of Medicine, Baltimore, MD USA; 7 Population Health Sciences Institute, Faculty of Medical Sciences, Newcastle University, Newcastle upon Tyne, UK; 8 Division of Cardiovascular Sciences, Faculty of Biology, Medicine and Health, University of Manchester, Manchester, UK; 9 Human Genetics Center; Department of Epidemiology, Human Genetics, and Environmental Sciences, UTHealth School of Public Health, Houston, TX USA; 10 Division of Cardiology, Children’s Hospital of Philadelphia, Philadelphia, PA USA; 11 Department of Pediatrics, Perelman School of Medicine, University of Pennsylvania, Philadelphia, PA USA; 12 Department of Surgery, Yale School of Medicine, New Haven, CT USA; 13 The Ward Family Heart Center, Section of Cardiac Surgery, Children’s Mercy Hospital, Kansas City, MO USA; 14 College of Biosciences, Kansas City University of Medicine and Biosciences, Kansas City, MO USA; 15 Texas Children’s Hospital, Houston, TX USA; 16 Heart Center, Nationwide Children’s Hospital, Columbus, OH USA; 17 Departments of Pediatrics, Pathology, and Human Genetics, The University of Chicago, Chicago, IL USA; 18 Department of Pediatrics, Emory University School of Medicine, Atlanta, GA USA

**Keywords:** Genomic analysis, DNA sequencing, Genetic variation, Aneuploidy, Cardiovascular genetics, Congenital heart defects

## Abstract

Atrioventricular septal defects (AVSD) are a severe congenital heart defect present in individuals with Down syndrome (DS) at a > 2000-fold increased prevalence compared to the general population. This study aimed to identify risk-associated genes and pathways and to examine a potential polygenic contribution to AVSD in DS. We analyzed a total cohort of 702 individuals with DS with or without AVSD, with genomic data from whole exome sequencing, whole genome sequencing, and/or array-based imputation. We utilized sequence kernel association testing and polygenic risk score (PRS) methods to examine rare and common variants. Our findings suggest that the Notch pathway, particularly *NOTCH4*, as well as genes involved in the ciliome including *CEP290* may play a role in AVSD in DS. These pathways have also been implicated in DS-associated AVSD in prior studies. A polygenic component for AVSD in DS has not been examined previously. Using weights based on the largest genome-wide association study of congenital heart defects available (2594 cases and 5159 controls; all general population samples), we found PRS to be associated with AVSD with odds ratios ranging from 1.2 to 1.3 per standard deviation increase in PRS and corresponding liability r^2^ values of approximately 1%, suggesting at least a small polygenic contribution to DS-associated AVSD. Future studies with larger sample sizes will improve identification and quantification of genetic contributions to AVSD in DS.

## Introduction

Congenital heart defects (CHD) are present in over 40% of infants with Down syndrome (DS), with the vast majority being septal defects^1^. Among septal defects, atrioventricular septal defects (AVSD) are the most severe, requiring surgery early in life. Approximately 20% of those with DS have an AVSD, compared to only 1 in 10,000 in the non-DS population^2^. This > 2000-fold increase in AVSD prevalence strongly suggests that trisomy 21 and resulting dysregulation of the genome substantially increase the risk for this disorder. Furthermore, it is likely that other genetic variation across the genome contributes to DS-associated AVSD; however, identification remains elusive. Efforts to clarify the genetic basis of AVSD in DS are important, as improved understanding of genetic causes may inform future work that facilitates a decrease in AVSD burden among the DS community; moreover, it has potential to shed light on fundamental biology relevant to the formation of CHD generally, which could yield benefits that extend beyond those with DS.

There have been several studies of the role of common variants in DS-associated AVSD, including the largest genome-wide association study (GWAS) to date with 210 complete AVSD cases with DS (DS + AVSD) and 242 controls with DS and structurally normal hearts (DS + NH). These studies have not identified any common variants (single nucleotide polymorphisms [SNP] or copy number variants [CNV]) exceeding genome-wide significance, despite adequate sample sizes for detecting common variants with large effect sizes^3–5^. This suggests that large-effect common variants (e.g., odds ratios > 2.0) do not play a considerable role in DS-associated AVSD. However, low to moderate-effect common variants including SNPs and structural variants may be contributing to risk, perhaps in a cumulative way^6^.

Rare variant studies of AVSD have yielded some positive results, both among those with DS and those with non-syndromic AVSD. In a targeted sequencing study of 26 AVSD candidate genes among 141 DS + AVSD cases and 141 DS + NH controls, rare variants with predicted deleterious effects were found to be enriched in cases for genes involved in the vascular endothelial growth factor pathway^7^. A CNV analysis of the aforementioned 210 DS + AVSD cases and 242 DS + NH controls identified a suggestive enrichment of large rare deletions in ciliome genes among cases^3^. A more recent study of 198 DS + AVSD cases and 211 DS + NH controls (a subset of those analyzed by Ramachandran et al.^3,4^) investigated CNVs on the trisomic chromosome 21. The investigators found controls self-identifying as African-American to have more bases covered by rare deletions than African-American cases, while cases self-identifying as Caucasian had more genes intersected by rare duplications than Caucasian controls^5^.

Among those with nonsyndromic AVSD, a rare variant exome study (minor allele frequency [MAF] < 0.01) involving 13 parent–offspring trios of probands and 112 unrelated controls revealed cases to be enriched for missense variants in *NR2F2*^8^, a gene that encodes for a nuclear receptor that is part of a steroid hormone superfamily and has been shown to play a role in heart development in mouse studies^9,10^. These findings suggest that rare variants may play an important role in DS-associated AVSD, warranting further study with larger sample sizes.

The limited success in identifying AVSD-associated sequence variants, both common and rare, may be due to small sample sizes that inhibit discovery. It is also possible that the genetic architecture of CHD is more complex than originally hypothesized. For common complex disorders such as schizophrenia and cardiovascular disease, it is now understood that there is a polygenic component to risk, whereby hundreds or thousands of common variants each incrementally increase risk for the phenotype^11,12^. AVSD may similarly have a polygenic component contributing to risk, which could be especially relevant when combined with a genomic background in which many genes are dysregulated due to trisomy 21. This polygenic component can be quantified using a polygenic risk score (PRS) methodology, which examines the extent to which common variants (MAF > 0.05) may be collectively contributing to a phenotype.

Rare variants may also play an important role in AVSD. When working with a rare disorder such as DS + AVSD, it is essential to maximize the power of small sample sizes, as it is anticipated that many mutations will be private or ultra-rare. This notion supports the use of burden tests or the sequence kernel association test (SKAT)^13^, which group rare variants into those occurring in genes or pathways. Use of the optimal unified test (SKAT-O) maximizes the advantages of both types of combined variant testing by modeling both SKAT and the burden test for each defined variant set and finding the optimal linear combination of both tests, thus optimizing power for the test^14^. SKAT-O can be employed for analysis of common variants within genes and pathways as well as for rare variant testing^15^.

While the PRS and SKAT-O approaches are not designed to pinpoint individual genetic variants as associated with the target phenotype, they provide insight into the genetic underpinnings of the trait of interest that complements standard analyses of individual genetic variation. With this in mind, we implemented the PRS approach and SKAT-O in order to optimally examine whether common and rare variants may be contributing to DS-associated AVSD and to identify those genes and pathways that may be most relevant to this phenotype.

## Methods

### Subjects

Participant samples for the whole exome sequencing (WES) and whole genome sequencing (WGS) studies that were defined as “cases” were obtained through three projects: the Down Syndrome Heart Project (DSHP), the Pediatric Cardiac Genomic Consortium (PCGC), and a consortium of collaborative surgical centers. The DSHP is a multi-site project conducted in the United States (details described in Ramachandran et al. 2015 and Ramachandran et al. 2015)^3,4^. PCGC case probands were collected from multiple sites in the United States and United Kingdom as described in Hoang et al. 2018 and Hoang et al. 2013^16,17^. The PCGC parent study recruited probands with heart defects, and in these analyses, we only used data from those who also had trisomy 21. Cases from the DSHP (n = 252) and from the PCGC (n = 47) were defined as individuals with trisomy 21 with a complete, balanced AVSD diagnosed by echocardiogram or surgical reports. For the surgical collaborative, case probands were ascertained from those referred for operative repair. Again, all case probands had trisomy 21; 37 cases were diagnosed with complete AVSD, 19 with AVSD-Not otherwise specified, two with AVSD + Tetralogy of Fallot, and two with single ventricle/unbalanced AVSD. We refer to all cases as DS + AVSD cases.

“Controls” were only drawn from the DSHP, and were defined as individuals with trisomy 21 and a structurally normal heart, patent foramen ovale, or patent ductus arteriosus. The majority of controls were defined based on echocardiograms. We refer to all controls as DS + NH controls.

The WES dataset was used in the primary gene-set analyses and included case proband data from a subset of those ascertained from DSHP as well as all DS cases ascertained through the surgical collaborative. The WGS dataset was used as follow-up of the WES findings and included data from the remaining DSHP cases and all of the PCGC case samples. There was no overlap in samples in the WES and WGS analytic datasets. Table 1 provides the sample sizes of the WES and WGS datasets.

**Table 1:** Summary of cohort for SKAT-O analyses.

	WES dataset		WGS dataset	
	Cases	Controls	Cases	Controls
Total	190 (174)	138 (126)	169 (148)	39 (27)
**Sex**				
Male	81 (75)	87 (79)	74 (66)	23 (17)
Female	109 (99)	51 (47)	95 (82)	16 (10)
**Ethnicity**				
Caucasian	152 (152)	101 (101)	161 (148)	35 (27)
African American	34 (18)	37 (25)	7 (0)	4 (0)
Ad-mixed American	2 (2)	0	0	0
East Asian	2 (2)	0	0	0
Hispanic	0	0	1 (0)	0

Total cohort numbers after quality control and principal component analysis used for SKAT-O analyses are in parentheses.

All experimental protocols for this study were approved by the Institutional Review Boards (IRB) of Emory University (Atlanta, GA); Johns Hopkins University and Kennedy Krieger Institute (Baltimore, MD); Oregon Health and Science University (Portland, OR); and all other participating universities and clinical sites. The authors assert that all methods used in this study were carried out in accordance with the relevant guidelines and regulations: all procedures contributing to this work comply with the ethical standards of the relevant national and institutional committees on human experimentation and with the Helsinki Declaration of 1975, as revised in 2008. Informed consent was obtained from the parent or guardian of each participant before assessments were completed and biological samples obtained.

#### Whole exome sequencing

WES was performed on 190 DS + AVSD cases and 138 DS + NH controls by the National Heart, Lung, and Blood Institute’s Resequencing and Genotyping Service at the University of Washington. FASTQ files from single-ended WES were mapped and variants were called with Emory’s PEMapper and PECaller, respectively^18^. Variants were annotated using Bystro (https://bystro.io)^19^. A total of 331,935 single nucleotide variants (SNVs) were detected by WES across the 190 cases and 138 controls. Mean coverage depth ± standard deviation (sd) of exome sequencing was 1.57 ± 0.39 for samples and mean transition/transversion ratio ± sd was 2.7 ± 0.15.

Sample failures were addressed by removing any individuals missing > 1% genotypes or failing PLINK1.9’s sex check (based on F statistics for X chromosome heterozygosity, which were also used to impute sex on individuals missing sex data)^20–22^. These filters identified no samples for exclusion. Variant filters included removing SNVs with missingness > 10% and those failing the exact test for Hardy–Weinberg equilibrium (HWE) at a *p* value < 10^–6^.

We then performed principal component analysis (PCA), using PLINK1.9. We used common SNPs (MAF > 0.05) and pruned SNPs in linkage disequilibrium with an r^2^ > 0.2, stepping along five SNPs at a time within 50-kilobase windows. Through three rounds of PCA we identified a total of 28 outlier samples for removal. Following quality control (QC), the WES dataset contained 300 samples (174 cases, 126 controls) and 330,287 SNVs for analysis.

#### Whole genome sequencing

Paired-ended WGS was performed on 169 DS + AVSD cases and 39 DS + NH controls by Hudson Alpha (Huntsville, AL) to a target depth of 30 ×. Raw FASTQ data were mapped and variants were called using PEMapper and PECaller, respectively, and variants were annotated using Bystro. In total, 12,302,231 SNVs were detected by WGS across 169 cases and 39 controls. Mean coverage depth ± SD was 30.2 ± 4.1. Mean transition/transversion ratio ± SD was 2.05 ± 0.007.

Sample failures were addressed by removing samples with theta < 3 sd below mean theta, transition/transversion ratio < 3 sd below mean transition/transversion ratio, heterozygosity/homozygosity ratio > 4 sd above mean heterozygosity/homozygosity ratio, missing > 1% of genotypes, or failing PLINK1.9’s sex check, and excluding one sample from each pair of related samples (with related samples defined as having a proportion of alleles shared identical by descent [IBD] > 0.1875). These steps resulted in the removal of 16 poor quality WGS samples. We also excluded one WGS sample identified as a duplicate of a WES sample. Variant QC involved removing SNVs with missingness > 10%, and those failing the exact test for HWE among cases and controls combined at a *p* value < 10^–12^.

We performed PCA in the same manner as described for the WES dataset. Three rounds of PCA identified a total of 16 additional WGS samples as outliers for removal. After these QC steps, the WGS dataset contained 175 samples (148 cases and 27 controls) and 12,279,101 variants.

#### Samples with imputed genotypes based on microarray

Affymetrix Genome-Wide Human SNP 6.0 array genotype data were available for 459 DS samples (211 DS + AVSD cases, 248 DS + NH controls), including 198 (100 cases, 98 controls) of the 328 WES samples and 95 (all cases) of the 208 WGS samples described above. Array data for these 459 DS samples were originally generated and analyzed in the prior GWAS and CNV analysis of DS-associated AVSD^3,4^. We applied standard GWAS QC and PCA procedures to these data (see Supplementary Methods for details) using PLINK1.9 and R (version 3.4.1)^23^, which yielded a dataset with 207 cases and 234 controls, all of European ancestry, and 612,125 autosomal SNPs (excluding the trisomic chromosome 21).

For these samples, we performed genotype imputation using the Michigan Imputation Server^24^. Genotype imputation was based on the Haplotype Reference Consortium (HRC) panel (version r1-1 2016)^25^, which includes 32,470 samples predominantly of European ancestry. The post-imputation files included 38,596,402 autosomal variants (all SNPs). Mean correlation between true and imputed genotypes for the ~ 600,000 genotyped SNPs was 0.990, suggesting high quality imputation. For this dataset (referred to as the “imputed dataset”), we excluded variants with MAF < 0.01, those missing for more than 2% of samples, and those with imputation r^2^ < 0.80; and we set as missing any genotypes with maximum imputed genotype probability < 0.80.

We then applied standard GWAS QC to the imputed dataset (see Supplementary Methods for details). We also removed variants with A/T, T/A, C/G, and G/C alleles which can be difficult to match between datasets due to strand ambiguity; this was done in preparation for merging this imputed dataset with unique WGS samples, to create a larger sample for the PRS analyses (these steps are explained in detail in the PRS Analysis section). This left an imputed dataset with 440 samples (206 cases, 234 controls) and 5,079,537 autosomal SNPs.

### Analyses

#### SKAT-O variant analyses

All variants in both the WES and WGS datasets were filtered using Bystro to include only exonic and untranslated regions (UTR) of the genome for any transcript as defined by the National Center for Biotechnology Information’s Reference Sequence Database (RefSeq) for human genome build 38 (hg38). Variants in multiple overlapping genes were included in each gene separately. They were further filtered using Bystro to only include SNVs. Separate analyses were conducted for variants within the specified MAF categories (Table 2). Genome Aggregation Database (gnomAD) MAFs were used to define eligible variants for rare or common analyses. In order to capture variants at a very low population MAF in our dataset, variants missing from gnomAD were included in the rare analysis. As an additional step to ensure those missing variants were truly at a low MAF, the variants that were missing allele frequency information in gnomAD were filtered based on the WES or WGS dataset at MAF < 0.02. In contrast, for the ultra-rare analysis we excluded the variants missing from gnomAD in order to test whether well-defined ultra-rare variants could be driving the top rare results. MAFs from gnomAD were used as weights in the rare and ultra-rare variant analyses (Table 2). Records where the reference allele was the minor allele were excluded. Variants in chromosome 21 were excluded. SKAT-O testing was done using the SKAT package in R using sex and the first five principal components of ancestry as covariates. For the SKAT-O analysis, the WES dataset was analyzed first. Genes with a resulting *p* < 0.001 (small-sample adjusted) were then evaluated in the WGS dataset by SKAT-O. The genes with the lowest *p* value were evaluated as candidate genes. Candidate genes were checked for cardiac phenotypes and heart expression using data from the Genotype-Tissue Expression (GTEx) project.

**Table 2:** Summary of genes analyzed using SKAT-O, based on variants in exons and UTRs.

SKAT-O analysis	MAF filter	Genes	SNVs	MAF weighting in SKAT
WES-common	MAF > 0.05	10,228	25,355	No
WES-rare	MAF < 0.01 and missing in gnomAD	17,318	142,006	Yes
WES-ultra-rare	MAF < 0.001	14,898	59,092	Yes

#### Rare variant pathway analyses

As a follow-up to the gene-based SKAT-O tests, two pathways that our top candidate genes belong to were evaluated due to their reported involvement in heart development: (1) ciliome gene set^26^ and (2) Notch pathway^27^. Each was evaluated as a single gene set. For the ciliome, 3573 SNVs identified in 301 genes found in the van Dam Ciliome gene list were evaluated^26^. For the Notch pathway, 487 SNVs identified in 48 genes in the Notch pathway defined by the Kyoto Encyclopedia of Genes and Genomes (KEGG) were analyzed^27^. All variants within a defined MAF-filtered category (common, rare, and ultra-rare) were analyzed with SKAT-O.

#### Polygenic Risk Score analyses

We have grouped the PRS analyses into primary and secondary analyses. The primary analyses had the goal of examining the genome-wide polygenic contribution to DS-associated AVSD, while the secondary analyses had the goal of estimating the additional polygenic contribution specifically due to the trisomic chromosome 21. These primary and secondary PRS analyses utilized slightly different target datasets and slightly different processes for generating and analyzing the PRS (as described below), but employed the same discovery datasets for weighting alleles in the PRS.

#### Target dataset for primary PRS analyses

The target sample for our primary PRS analyses included 245 DS + AVSD cases and 242 DS + NH controls and represents a combination of the WGS and imputed datasets. We prepared the WGS dataset (175 samples) for merger with the imputed dataset (440 samples) by removing variants with MAF < 0.01, those missing for > 2% of samples, and indels (filters which had already been applied to the imputed dataset). We then merged these datasets and subjected the resulting 615 samples and 2,366,788 SNPs to standard QC measures. An IBD check identified 90 sample duplicates and 1 sample pair with a sibling or child/parent relation. Each of these related pairs involved a WGS sample and an imputed sample (i.e., the duplicates were the result of each sample being represented in both the imputed and WGS datasets). For these samples, we kept the data from the WGS dataset as it appeared to be of slightly better quality overall, and we dropped the imputed duplicates. No additional variants required removal. Note that because imputed data were not available for the trisomic chromosome 21 (methods for imputing trisomic genotypes are lacking), this target dataset for the primary analyses did not include chromosome 21 variants. This intermediate data set included 524 samples (263 cases, 261 controls) and 2,366,788 autosomal SNPs.

We then performed PCA, first anchoring our dataset in the HapMap3^28^ dataset and constructing principal components (PC) using PLINK1.9 in order to identify and remove DS samples with PC values outside of the HapMap3 CEPH/Utah (CEU) cluster (in order to match the European ancestry of the discovery datasets), and then removing the HapMap samples and performing further outlier removal based only on the DS samples (see Supplementary Methods for details). This PCA process identified 37 sample outliers for removal.

As a final step in preparing the DS target dataset for PRS analysis, we removed the extended major histocompatibility complex region (chromosome 6, ~ 25,000,000–34,000,000, human genome build 19), which is a region of extended high linkage disequilibrium that can overly influence PRS results. Our final data set included 487 samples (245 DS + AVSD cases, 242 DS + NH controls) and 2,351,951 autosomal SNPs (excluding chromosome 21). The multiple steps involved in generating this final data set for the primary PRS analyses are presented as a flowchart in Supplementary Fig. S1.

#### Target dataset for secondary PRS analyses

Our secondary PRS analyses examined the contribution by alleles on the trisomic chromosome 21 to a polygenic component for DS-associated AVSD. We were able to do this because, while imputed data were not available for chromosome 21, all imputed samples did have SNP array genotypes for chromosome 21. Furthermore, the WGS samples had sequencing data for chromosome 21. For all target samples analyzed in the primary analyses (245 cases, 242 controls), we therefore obtained SNP-array-level data for the trisomic chromosome 21, and likewise limited all other chromosomes to SNPs available on the Affymetrix Genome-Wide Human SNP 6.0 array.

We processed the chromosome 21 data separately from the other chromosomes due to the trisomic nature of these data. Prior to merging chromosome 21 data for the imputed and WGS samples, we applied certain QC filters (see Supplementary Methods for details). We subsequently merged the array and WGS chromosome 21 data, leaving 3984 chromosome 21 SNPs and 487 samples.

#### Discovery data used to define weights for the PRS

For discovery datasets, there were no GWAS of AVSD or other congenital heart defects (CHD) among individuals with DS that were independent of our target dataset nor were there any GWAS specifically for non-syndromic AVSD. Thus, we used results from two of the largest available independent GWAS of mixed CHD, diagnosed among those without DS who were ancestrally matched to our target samples.

The first discovery dataset was a GWAS of 2594 cases with a mixture of CHD diagnoses (see Table 3) and 5159 population-based controls, all of European ancestry. Genotyping was performed using the Illumina Human660W-Quad array for cases and the Illumina 1.2 M chip for controls. The GWAS results included summary statistics for 501,899 autosomal SNPs. Summary level results for this GWAS are available upon request through Dr. Heather Cordell. GWAS of particular diagnostic CHD subsets of this dataset have been published previously^29,30^.

**Table 3:** First discovery dataset: diagnoses for 2594 mixed CHD cases^29,30^.

CHD diagnosis	Number (%) of samples
Tetralogy of Fallot	835 (32.2)
Left-sided malformations	387 (14.9)
Ostium secundum atrial septal defect	340 (13.1)
Transposition of the great arteries	207 (8.0)
Ventricular septal defect	191 (7.4)
Conotruncal malformations	151 (5.8)
Double outlet right ventricle	96 (3.7)
AVSD (partial and complete)	73 (2.8)
Other CHD*	314 (12.1)

*For a more complete list of included CHD diagnosis, see^29,30^.

The second discovery dataset was a GWAS of 406 mixed CHD cases (Table 4) and 2976 pediatric controls, all recruited from the same hospital and self-reporting as non-Hispanic Caucasian^31^. Samples were genotyped with Illumina arrays (550 v1/v3, 610, or 2.5 M chip), and genome-wide imputation was then carried out using the 1000 Genomes Project data as a reference. The GWAS results included summary statistics for 4,612,359 autosomal SNPs, all of which had imputation r^2^ > 0.80. Summary results from this GWAS are available upon request through Dr. A.J. Agopian.

**Table 4:** Second discovery dataset: diagnoses for 406 mixed CHD cases^31^.

CHD diagnosis	Number (%) of samples
Tetralogy of Fallot	134 (33.0)
Ventricular septal defect	109 (26.8)
D-transposition of the great arteries	80 (19.7)
Double outlet right ventricle	25 (6.2)
Isolated aortic arch anomalies	22 (5.4)
Truncus arteriosus	19 (4.7)
Other CHD	17 (4.2)

We used each of these discovery datasets separately as training data for the PRS analyses. We also meta-analyzed the summary results from these two GWAS using Genome-Wide Association Meta-Analysis (GWAMA) software^32^, and used the resulting estimates as training data.

#### Generating PRS for the primary analyses

For the primary PRS analyses, PRSice-2 (version 2.1.6)^33^ was used to generate PRS for each sample in the target dataset. Prior to PRS construction, PRSice performs clumping on the discovery dataset in order to obtain a set of independent SNPs for scoring (clumping parameters: 500-kilobase window, r^2^ threshold 0.10). The clumped SNPs are then used to generate PRS, which are calculated as

$${PRS}_{j}= \sum_{i}\frac{{\beta }_{i} \times {EA}_{ij}}{{N}_{j}}$$

where the subscript *i* denotes a specific SNP contributing to the PRS, the subscript *j* denotes a particular individual in the target dataset, *β* is the estimated effect from the discovery GWAS (e.g., the natural logarithm of the odds ratio), *EA* is the number of effective alleles possessed by the target individual (0, 1 or 2 for a disomic chromosome), and *N* is the total number of alleles considered for scoring. To facilitate interpretation of results, we applied an option in PRSice to standardize the PRS.

We constructed multiple PRS for each target individual using different subsets of the set of clumped SNPs, with subsets determined by applying different *p* value thresholds to the discovery GWAS results (e.g., PRS may be constructed using SNPs with discovery *p* value < 1 × 10^–6^, < 0.05, < 1). Given the relatively small sample sizes for each discovery GWAS, we were concerned that effect estimates for SNPs with lower MAFs may be particularly subject to error. To address this, we applied a range of MAF filters (from 0.10 to 0.40) to the discovery datasets prior to generating the PRS, excluding those SNPs with MAF below the threshold. Thus, for each discovery dataset, we constructed PRS and performed separate analyses for each combination of MAF filter and discovery *p* value threshold.

#### Generating PRS for the secondary analyses

For the secondary PRS analyses, which involved analyses both with and without the trisomic chromosome 21 data, we constructed PRS using PLINK1.9. The PLINK1.9 binary, which is the file format that we used in conjunction with PRSice for the primary PRS analyses, is not able to represent trisomic genotype data. However, we were able to modify the chromosome 21 genotype data to fit the PLINK1.9 dosage file format, which can be used in conjunction with PLINK’s allelic scoring flag to generate PRS. This involved dividing each allele count by 3 and thereby converting allele counts of 0, 1, 2 and 3 to values of 0, 1/3, 2/3 and 1 (interpreted by PLINK as dosages ranging from 0 to 1). We then used this chromosome 21 dosage format file in combination with the clumped training data (clumped using PRSice) to generate PRS, which were generated by PLINK as a simple sum score (a sum of the products of SNP weight times transformed allele count for each scoring SNP). Finally, we multiplied each outputted PRS by 3, yielding PRS that accurately reflected allele counts of 0, 1, 2 and 3 for the trisomic chromosome 21.

Separately, we used PLINK1.9 to construct PRS for the remaining autosomes. Given that these remaining autosomes were diploid, we were able to use the standard PLINK1.9 binary in combination with the allelic scoring flag to generate PRS. To be consistent with the chromosome 21 PRS, we used an option to generate these PRS as sum scores. For the analyses including chromosome 21, we then summed the chromosome 21 PRS and the PRS for the remaining autosomes for each target individual, yielding a PRS based on alleles from all autosomes combined. The analyses excluding chromosome 21 only utilized the PRS based on all autosomes minus chromosome 21. As for the primary PRS analyses, we standardized the final PRS, and generated multiple PRS for each target individual based on different discovery GWAS *p* value and MAF thresholds.

#### Testing association of PRS with DS + AVSD

We used logistic regression to test associations of PRS with the outcome; this was performed by PRSice for the primary analyses and within R for the secondary analyses. We included sex, platform (WGS vs. imputed), and the top five principal components of ancestry as covariates in the analyses. Tests were two-tailed. Given the multiple testing involved in these PRS analyses (394 tests for different combinations of MAF filter, *p* value threshold, and discovery and target datasets, considering the primary and secondary PRS analyses together), we employed the *p* value adjusted for correlated tests (*P*_ACT_)^34^ method to generate *p* values corrected for multiple correlated tests.

## Results

### Gene discovery using SKAT analyses

Three separate SKAT-O analyses were conducted at different MAF filtering thresholds. All of the *p* values for SKAT-O analyses derived from the WES dataset fell within expected or slightly deflated values, likely due to the small sample size (Supplementary Fig. S2). No gene was significant following Bonferroni correction for the total number of genes in each set, although 19 genes in the common variant analysis (MAF > 0.05), 10 genes in the rare variant analysis (MAF < 0.01 or missing in gnomAD) and one gene in the ultra-rare variant analysis (MAF < 0.001) displayed nominal significance levels of *p* value < 10^–3^ (Tables 5, 6, 7). Of those genes with nominal significance in the WES dataset, three were supported in the WGS dataset. Two of those genes, *NOTCH4* and *CEP290*, have been reported as being involved in heart development. *NOTCH4* is expressed in the developing heart and has previously been identified as playing a role in early artery and endothelial-to-mesenchymal transformation, which is critical for endocardial cushion differentiation^35,36^. *CEP290* codes for a centrosomal protein involved in cilia development that has been found to have differential expression between the left and right ventricles of the heart in newborn piglets, and which may play a role in remodeling of the ventricular myocardium postnatally^37^. The third gene, *ZNF318*, has not previously been implicated in heart defects.

**Table 5:** SKAT-O results of common variants: common variants are defined as MAF > 0.05 in gnomAD.

Gene	Locus	*p* value—WES	Variants tested	*p* value—WGS	Variants tested
*AMOT*	chrX:112,774,503–112,840,815	8.21E−04	1	0.849	6
*CEP290*	chr12:88,049,016–88,142,088	1.88E−04	3	0.064	3
*CTDSP1*	chr2:218,399,755–218,405,941	8.82E−04	2	0.392	3
*DNAJA4*	chr15:78,264,086–78,282,196	2.37E−04	2	0.296	7
*GABRE*	chrX:151,953,124–151,974,676	2.49E−04	1	0.339	6
*HHAT*	chr1:210,328,252–210,676,296	6.39E−04	3	0.576	5
*HJURP*	chr2:233,836,702–233,854,535	8.49E−04	7	0.592	10
*MEFV*	chr16:3,242,028–3,256,627	7.18E−04	8	0.388	12
*MRGPRX3*	chr11:18,120,955–18,138,480	6.07E−04	3	0.808	6
*MYO5B*	chr18:49,822,789–50,195,147	2.72E−04	5	0.263	14
*NEK10*	chr3:27,110,904–27,369,392	8.52E−04	3	0.092	8
NR0B2	chr1:26,911,489–26,913,975	5.91E−04	1	0.83	3
*PLEKHM3*	chr2:207,821,288–208,025,527	3.48E−04	2	0.826	10
*SAG*	chr2:233,307,816–233,347,055	3.35E−04	3	0.527	3
*TRMT9B*	chr8:12,945,673–13,029,777	6.22E−05	8	0.627	40
*WDR61*	chr15:78,283,235–78,299,609	5.98E−04	1	0.414	2
*WDR87*	chr19:37,884,932–37,906,677	8.81E−05	4	0.198	13
*ZNF571*	chr19:37,562,392–37,594,790	8.60E−04	3	0.203	5
*ZNF573*	chr19:37,738,302–37,779,590	7.08E−04	2	0.148	3

All genes were tested in the WES dataset; only the top-ranked genes (*p* < 0.001) were tested in the WGS dataset as a replication set. Common variant SKAT-O analyses were not weighted by MAF. In the ‘Locus’ column, ‘chr’ refers to chromosome.

**Table 6:** SKAT-O results of rare variants: rare variants are defined as MAF < 0.01 or missing in gnomAD with an additional dataset MAF filter < 0.02.

Gene	Locus	*p* value—WES	Variants tested	*p* value—WGS	Variants tested
*ALG11*	chr13:52,012,398–52,033,600	9.66E−04	6	0.543	6
*CST4*	chr20:23,685,640–23,689,040	6.21E−04	8	0.815	5
*NOTCH4*	chr6:32,194,843–32,224,067	6.66E−04	9	0.031	10
*PODNL1*	chr19:13,933,957–13,953,302	7.86E−04	5	0.568	14
*RBM12*	chr20:35,648,925–35,664,900	9.04E−04	3	0.523	8
*RNF135*	chr17:30,971,039–30,999,911	9.14E−04	6	0.651	5
*RNF152*	chr18:61,808,067–61,893,007	5.06E−04	7	0.701	7
*SLIT3*	chr5:168,661,740–169,301,129	7.30E−04	23	0.484	23
*TRIM56*	chr7:101,085,481–101,097,967	4.73E−04	8	0.836	6
*VCX3A*	chrX:6,533,618–6,535,118	9.07E−04	4	0.69	4

All genes were tested in the WES dataset; only the top-ranked genes (*p* < 0.001) were tested in the WGS dataset as a replication set.

**Table 7:** SKAT-O results of ultra-rare variants: ultra-rare variants are defined as MAF < 0.001 in gnomAD without variants missing in gnomAD to test whether the well-defined ultra-rare variants are driving the top rare results.

Gene	Locus	*p* value—WES	Variants tested	*p* value—WGS	Variants tested
*ZNF318*	chr6:43,336,070–43,369,647	8.07E−04	17	0.042	2

All genes were tested in the WES dataset; only the top-ranked genes (*p* < 0.001) were tested in the WGS dataset as a replication set.

As a follow-up of these results, we conducted gene-set analyses based on genes in the ciliome and those in the Notch pathway using the WES dataset. We used SKAT-O, combining all variants identified in each pathway and again filtering on MAF thresholds, and found moderate significance of *p* < 0.05 in the set of rare variants (MAF < 0.01 or missing in gnomAD) (Table 8).

**Table 8:** SKAT-O results in WES dataset for the two pathways suggested by the single gene test results and by previous literature.

Gene	Common variants		Rare variants		Ultra-rare variants	
	*p* value	Variants tested	*p* value	Variants tested	*p* value	Variants tested
Cilia pathway	0.77	674	0.04	3542	0.80	1490
Notch pathway	0.24	73	0.03	487	0.39	222

### CHD polygenic risk score and its association with DS + AVSD

#### Primary analyses indicate a non-significant association of the CHD-based PRS with DS + AVSD

**Figure 1:**
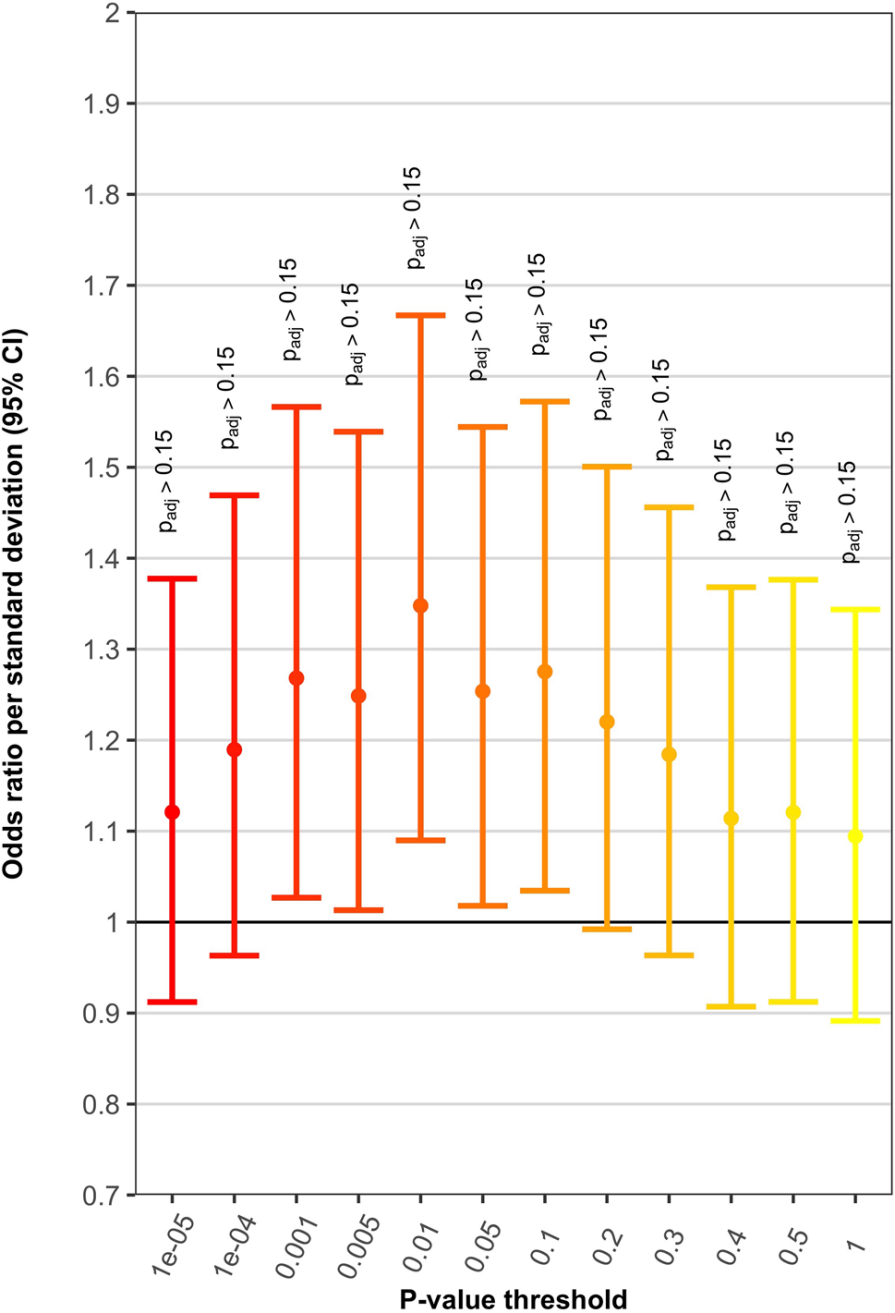
PRS results using discovery GWAS of 2594 mixed CHD cases and 5159 controls and SNPs with MAF ≥ 0.35. Plot shows odds ratio per standard deviation increase in PRS, with corresponding 95% confidence interval (CI). ‘*P* value threshold’ indicates that SNPs with discovery GWAS *p* values below the threshold were used for PRS construction. P_adj_ is the *p* value after correction for multiple correlated tests.

Over a range of MAF filters and discovery GWAS *p* value thresholds for constructing PRS, the analyses using the GWAS of 2594 mixed CHD cases and 5159 controls as the discovery dataset (501,899 autosomal SNPs) tended to yield maximum odds ratios (ORs) of 1.2–1.3 for association of PRS with AVSD among those with DS, meaning that a 1 standard deviation increase in PRS was associated with a 20–30% greater odds of having AVSD in the DS target sample (Supplementary Fig. S3). Corresponding Nagelkerke’s r^2^ values ranged from 0.75 to 1.25% (calculated as Nagelkerke’s r^2^ for the model with PRS and covariates minus Nagelkerke’s r^2^ for the model with only covariates), with *p* values that were non-significant following adjustment for multiple correlated tests (adjusted *p* values > 0.15; unadjusted *p* values approximately 0.01–0.09). These maximum results were most evident at higher MAF filters (i.e., MAF ≥ 0.30, ≥ 0.35, ≥ 0.40) and discovery GWAS *p* value thresholds between 0.001 and 0.3. Figure 1 and Supplementary Table S1 present results when PRS are constructed using SNPs with MAF ≥ 0.35, which are representative of the maximum PRS results achieved when using this particular discovery dataset.

PRS results when using the GWAS of 406 CHD cases and 2976 pediatric controls as the discovery dataset (4,612,359 autosomal SNPs) exhibited a different pattern than when using the GWAS of 2594 mixed CHD cases and 5159 controls as training data. Across various MAF filters and *p* value thresholds, ORs tended to hover near the null and on both sides of the null, indicating that these PRS were minimally associated with AVSD (Supplementary Fig. S4). A few results were stronger, with ORs in the 1.2–1.3 range (adjusted *p* values > 0.15); these results occurred when using MAF filters of ≥ 0.10 and ≥ 0.15 in combination with the smallest discovery GWAS *p* value thresholds for selecting scoring SNPs.

We also performed a meta-analysis of the two GWAS datasets, yielding a single discovery dataset with association estimates for 4,684,854 autosomal SNPs, of which 429,336 SNPs had estimates based on both studies (meta-analysis sample size of 3000 CHD cases and 8135 controls), while the remainder had estimates based on just one of the two studies. In constructing PRS based on this meta-analysis discovery dataset, we applied an inverse variance weighting approach such that SNP association estimates based on a larger sample size (e.g., two studies) were weighted more heavily. Using the meta-analysis dataset in this manner produced results which, as might be expected, were a mixture of the PRS results obtained when using each discovery GWAS dataset separately (Supplementary Fig. S5). In general, maximum ORs for association of AVSD in DS with PRS and corresponding Nagelkerke’s r^2^ values were slightly attenuated compared with results when using the GWAS of 2594 mixed CHD cases and 5159 controls as the discovery dataset.

#### Adding data from chromosome 21 into the PRS calculation did not change the association with DS + AVSD

We performed the secondary analyses using only the training dataset derived from 2594 mixed CHD cases and 5159 controls, since using these training data produced the best results for the primary PRS analyses. The results from PRS analyses including and excluding chromosome 21 were essentially the same, with only slight fluctuations in ORs and corresponding Nagelkerke’s r^2^ values (Supplementary Figs. S6 and S7). These results generally followed a similar pattern to those observed for the primary PRS analysis using the same discovery dataset (Supplementary Fig. S3), wherein use of greater MAF filters yielded larger associations. However, the results from these secondary analyses fluctuated more across discovery GWAS *p* value thresholds and included more outlier OR estimates, which was likely a result of the smaller number of SNPs used for scoring in the secondary analyses (which were limited to SNPs on the Affymetrix array).

## Discussion

Previous studies of AVSD in DS have had limited success in identifying rare variant contributions and have failed to clarify the role of common variants^3–7^. In the current study, we examined the role of rare and common SNVs in DS-associated AVSD by analyzing data from whole exome sequencing, whole genome sequencing, and genome-wide SNP imputation in cases with DS + AVSD and DS + NH controls. We used SNV-set analyses (grouping variants into genes and pathways) to examine both rare and common variant associations and polygenic risk score methods to investigate the combined effect of common variants across the genome.

In the genome-wide variant-set analyses which grouped SNVs by gene, we obtained preliminary support for 3 genes, 2 of which have been reported previously as genes involved in heart development. Prior studies in AVSD and other heart defects have identified cilia as a major factor in heart development^38,39^ and the ciliome has been identified as a pathway enriched in DS + AVSD for rare deletions and differential gene expression^3,40^. In our analyses, *NOTCH4* and *CEP290*, whose roles in heart development and the ciliome have been previously described^36,37^, were found to have nominally significant associations with DS + AVSD in both WES and WGS datasets. *CEP290* was also recently identified as potentially associated with non-syndromic CHD (including any type of heart defect) by a targeted sequencing study of 406 candidate genes involved in heart development^41^.

Investigating rare variants in the Notch pathway and ciliome genes as a whole also yielded moderate associations with DS + AVSD in our dataset. These results provide further support for the involvement of the Notch pathway and ciliome in heart development, and suggest a specific link between these pathways and AVSD in DS. Considering the small sample sizes of the WES and WGS datasets and the case–control imbalance in the WGS dataset, a larger balanced WGS dataset should enable greater power to detect individual genes in these pathways that contribute to AVSD in DS.

The PRS analyses are the first such analyses of AVSD in DS, and to the best of our knowledge they are also the first use of PRS methods to examine polygenicity of CHD generally. Our analyses of PRS calculated from GWAS studies of non-syndromic CHD suggest at minimum a small polygenic contribution to AVSD among individuals with DS. When using dense SNP data (WGS or imputed data) for the 487 individuals in the target sample and excluding chromosome 21, a single standard deviation increase in PRS was associated with a 20–30% increased odds for having AVSD, with Nagelkerke’s r^2^ values for PRS of around 1% (Fig. 1; Supplementary Fig. S3); this occurred when using the larger of the two independent discovery datasets. Assuming a population prevalence of 20% for AVSD among those with DS, these Nagelkerke’s r^2^ values are quite similar to the corresponding liability scale r^2^ values (correcting for case–control ascertainment). For instance, the PRS analyses depicted in Fig. 1 yielded a Nagelkerke’s r^2^ of 1.03% when applying MAF ≥ 0.35 and discovery GWAS *p* value ≤ 0.001 thresholds; the corresponding liability r^2^ estimate is 1.11%^42^. As demonstrated by the PRS results presented in Supplementary Figs. S6 and S7, which involved the use of array SNPs only, inclusion of dense genotype data for chromosome 21 is unlikely to substantially alter these estimates for the association of PRS with DS-associated AVSD; SNPs on chromosome 21 are perhaps not a key factor driving AVSD in DS.

Given the small sample sizes for the discovery GWAS datasets and prior research demonstrating that variance explained by PRS tends to increase as discovery GWAS sample size increases^11^, which is attributable to increased accuracy of the SNP effect estimates used as weights for the PRS, it seems likely that the use of a larger discovery GWAS of CHD will uncover a greater polygenic contribution to AVSD in DS. Furthermore, use of a large discovery GWAS that only includes the particular CHD subtypes which are most closely genetically related to AVSD (perhaps a GWAS including only AVSD and septal defect cases) may reveal a polygenic contribution to DS-associated AVSD that exceeds what we have identified. We demonstrate this in Supplementary Fig. S8, showing that under reasonable assumptions, using a discovery GWAS of phenotypes that are highly genetically correlated with the target phenotype (AVSD) will result in PRS r^2^ values that increase as discovery GWAS sample size increases; the discovery samples similar in size to those used for the current PRS analyses are only able to capture a portion of the true polygenic component (plots generated using the ‘avengeme’ R package)^43^.

The finding of an association of AVSD in DS with PRS constructed based on SNPs identified as having some measure of association with CHD in mixed CHD samples suggests the possibility of genetic overlap between AVSD and various other subtypes of CHD. This is consistent with the potential for investigations of DS-associated AVSD to shed light on fundamental biology relevant to CHD more generally. To further examine this potential genetic overlap, including which CHD subtypes may have the greatest shared genetic architecture with AVSD, it will be important to utilize large GWAS datasets of specific CHD subtypes rather than a mixture of CHD types.

We observed that PRS constructed based on the discovery GWAS of 2594 mixed CHD cases and 5159 controls consistently yielded ORs > 1 (indicating, as expected, that increased PRS was associated with increased AVSD risk). In contrast, PRS constructed using the discovery GWAS of 406 CHD cases and 2976 pediatric controls yielded OR estimates generally quite close to the null, and on both sides of the null. One possible reason for this difference is that the smaller-sized discovery GWAS had more imprecisely estimated SNP associations, leading to less informative PRS. Another possibility is that particular CHD diagnoses included within the larger discovery GWAS may be more genetically related to AVSD in DS than the CHD diagnoses in the smaller discovery GWAS. Indeed, the larger GWAS included 73 cases with AVSD, while in the smaller GWAS there were only seven instances of AVSD (six of the cases with double outlet right ventricle also had AVSD, and a single case had tetralogy of Fallot with atrioventricular canal septal defect).

In conclusion, while our analyses yielded no statistically significant findings following multiple testing correction, the results suggest that rare variation in certain pathways and common variants acting through a polygenic component may play roles in increasing risk for AVSD among those with DS. The use of larger sample sizes, including a larger DS + AVSD/DS + NH sample as well as larger discovery GWAS samples for PRS construction, is important as it will enable greater power for identifying and quantifying rare variant and polygenic contributions. It is also possible that genetic effects on DS-associated AVSD are particularly pronounced in the presence of certain environmental factors. This could be investigated in future studies by examining environmental interactions with potentially involved genetic factors including variation in the Notch pathway and ciliome as well as PRS.

## Supplementary information

Supplementary information.

## Data Availability

Affymetrix Genome-Wide Human SNP 6.0 array genotype data are available for 437 DS samples (DS + AVSD cases and DS + NH controls) via the Gene Expression Omnibus [GEO] data repository, accession number GSE60607. Genotypes for some of the samples described in this paper were excluded from GEO due to privacy concerns. WES data is available on request. WGS data results from the PCGC samples can be accessed through the PCGC dbGaP study (https://www.ncbi.nlm.nih.gov/projects/gap/cgi-bin/study.cgi?study_id=phs001194.v2.p2).
