# Author Correction: Identifying genetic factors that contribute to the increased risk of congenital heart defects in infants with Down syndrome

**DOI:** 10.1038/s41598-021-94021-x

**Published:** 2021-07-20

**Authors:** Cristina E. Trevino, Aaron M. Holleman, Holly Corbitt, Cheryl L. Maslen, Tracie C. Rosser, David J. Cutler, H. Richard Johnston, Benjamin L. Rambo-Martin, Jai Oberoi, Kenneth J. Dooley, George T. Capone, Roger H. Reeves, Heather J. Cordell, Bernard D. Keavney, A. J. Agopian, Elizabeth Goldmuntz, Peter J. Gruber, James E. O’Brien, Douglas C. Bittel, Lalita Wadhwa, Clifford L. Cua, Ivan P. Moskowitz, Jennifer G. Mulle, Michael P. Epstein, Stephanie L. Sherman, Michael E. Zwick

**Affiliations:** 1 Department of Human Genetics, Emory University School of Medicine, 300 Whitehead Biomedical Research Building, 615 Michael St., Atlanta, GA 30322 USA; 2 Department of Epidemiology, Rollins School of Public Health, Emory University, Atlanta, GA USA; 3 Division of Cardiovascular Medicine and the Heart Research Center, Oregon Health and Science University, Portland, OR USA; 4 Sibley Heart Center Cardiology, Department of Pediatrics, Children’s Healthcare of Atlanta, Emory University, Atlanta, GA USA; 5 Kennedy Krieger Institute, Baltimore, MD USA; 6 Department of Physiology and the Institute for Genetic Medicine, Johns Hopkins University School of Medicine, Baltimore, MD USA; 7 Population Health Sciences Institute, Faculty of Medical Sciences, Newcastle University, Newcastle upon Tyne, UK; 8 Division of Cardiovascular Sciences, Faculty of Biology, Medicine and Health, University of Manchester, Manchester, UK; 9 Human Genetics Center; Department of Epidemiology, Human Genetics, and Environmental Sciences, UTHealth School of Public Health, Houston, TX USA; 10 Division of Cardiology, Children’s Hospital of Philadelphia, Philadelphia, PA USA; 11 Department of Pediatrics, Perelman School of Medicine, University of Pennsylvania, Philadelphia, PA USA; 12 Department of Surgery, Yale School of Medicine, New Haven, CT USA; 13 The Ward Family Heart Center, Section of Cardiac Surgery, Children’s Mercy Hospital, Kansas City, MO USA; 14 College of Biosciences, Kansas City University of Medicine and Biosciences, Kansas City, MO USA; 15 Texas Children’s Hospital, Houston, TX USA; 16 Heart Center, Nationwide Children’s Hospital, Columbus, OH USA; 17 Departments of Pediatrics, Pathology, and Human Genetics, The University of Chicago, Chicago, IL USA; 18 Department of Pediatrics, Emory University School of Medicine, Atlanta, GA USA

Correction to: *Scientific Reports* 10.1038/s41598-020-74650-4, published online 22 October 2020

Ivan P. Moskowitz was omitted from the author list in the original version of this Article.

The Author Contributions section now reads:

“C.E.T., A.M.H., M.E.Z., S.L.S., M.P.E., D.J.C., and J.G.M. were all involved in the conception and design of the work presented in this manuscript. C.E.T. and A.M.H. jointly drafted the manuscript, with all authors reviewing and providing feedback on the manuscript. Ascertainment of the samples and phenotype assessment of individuals with DS were accomplished through the efforts of S.L.S., C.L.M., T.C.R., K.J.D., G.T.C., R.H.R, P.J.G., J.E.O., D.C.B., L.W., C.L.C., I.P.M., and E.G. (sponsor of PCGC-approved ancillary project). H.R.J., D.J.C., and B.L.R. performed mapping and variant calling for the WES and WGS datasets, and together with C.E.T. performed QC of these data. C.E.T. performed the gene- and pathway-level (SKAT) analyses. H.C. and J.O. were involved in the initial phase of these datasets and helped to set the framework for these analyses. H.J.C., B.D.K., A.J.A., and E.G. generated the GWAS summary results used as training data for the PRS analyses. A.M.H. performed imputation of the array genotype data, and QC and analyses for the PRS application.”

The Acknowledgements section now reads:

“First and foremost, we want to thank the families and individuals with Down syndrome for contributing their time and effort towards this project. The DS Heart Project (R.H.R.) was a collaborative project supported through several National Institutes of Health awards: R01 HL092981-01A1 (M.E.Z.) and R01 HL083300 (R.H.R.) from NIH/National Heart, Lung, and Blood Institute; R01 HD38979 (S.L.S.) from NIH/National Institute of Child Health and Human Development and GM117946 (M.P.E.) from NIH/National Institute of General Medical Sciences. Whole exome sequencing services that were done as part of the DSHP were provided by the University of Washington, Department of Genome Sciences under U.S. Federal Government contract number HHSN268201100037C (D. Nickerson). Whole genome sequencing services were supported by the Emory Integrated Genomics Core (EIGC) and the Emory Integrated Computational Core (EICC), which are subsidized by the Emory University School of Medicine and are part of the Emory Integrated Core Facilities. Partial support to M.E.Z was also provided by the Georgia Clinical & Translational Science Alliance which is funded by the National Center for Advancing Translational Sciences of the National Institutes of Health under Award number UL1TR002378. The American Heart Association Western States Affiliate provided support to H. Corbitt (grant number 16PRE30190012). I.P. Moskowitz was supported by the following grants: R01 HL092153, R01 HL124836, and R01 HL126509. We thank Dr. Eleanor Feingold for providing the chromosome 21 genotype calls for the SNP Affymetrix dataset. We thank the Pediatric Cardiac Genomic Consortium (PCGC) for its contributions to this work, and recognize E.G. as sponsor of the PCGC-approved ancillary project. The PCGC provided samples for the WGS study which were collected under the auspices of the National Heart, Lung, and Blood Institute’s Bench to Bassinet Program (https://benchtobassinet.com). Support for the PCGC is provided through grants from National Heart, Lung, and Blood Institute (U01-HL098188, U01-HL098147, U01-HL098153, U01-HL098163, U01-HL098123, U01-HL098162). We would like to thank Marianne S. Hird at the PCGC for her help on this project throughout its administration. The Agopian et al. (2017) GWAS discovery dataset used for the PRS analyses was generated with support from an Institutional Development Fund to The Center for Applied Genomics from The Children’s Hospital of Philadelphia. Further support was provided by the National Heart, Lung, and Blood Institute P50-HL74731 (E.G.) and the National Center for Research Resources M01-RR-000240, RR024134 [now the National Center for Advancing Translational Sciences UL1TR000003) (E.G.). The Cordell et al. (2013) GWAS discovery data used for the PRS analyses were generated with support from the British Heart Foundation, Heart Research UK, Wellcome Trust and European Union. B.D.K. is supported by a British Heart Foundation Personal Chair. The content is solely the responsibility of the authors and does not necessarily represent the official views of the National Institutes of Health.”

The original Article and accompanying Supplementary Information file have been corrected.

