# Supplementary material for: Identifying genetic factors that contribute to the increased risk of congenital heart defects in infants with Down syndrome
Source: Sci Rep. 2020 Oct 22;10:18051. doi: 10.1038/s41598-020-74650-4 (PMC7582922; doi:10.1038/s41598-020-74650-4)
Supplement: Supplementary file 1 — Supplementary information. [file 41598_2020_74650_MOESM1_ESM.pdf]

Title:

Identifying genetic factors that contribute to the increased risk of congenital heart defects in infants with Down syndrome

Authors:
Cristina E. Trevino[1,†], Aaron M. Holleman[2,†], Holly Corbitt[3], Cheryl L. Maslen[3], Tracie C. Rosser[1], David J. Cutler[1], H. Richard Johnston[1], Benjamin L. Rambo-Martin[1], Jai Oberoi[1], Kenneth J. Dooley[4], George T. Capone[5], Roger H. Reeves[6], Heather J. Cordell[7], Bernard D. Keavney[8], A.J. Agopian[9], Elizabeth Goldmuntz[10,11], Peter J. Gruber[12], James E. O'Brien, Jr.[13], Douglas C. Bittel[14], Lalita Wadhwa[15], Clifford L. Cua[16], Ivan P. Moskowitz[17], Jennifer G. Mulle[1], Michael P. Epstein[1], Stephanie L. Sherman[1,18], Michael E. Zwick[1,18,*]
[†]Co-first authors

Affiliations:
[1] Department of Human Genetics, Emory University School of Medicine, Atlanta, GA, USA
[2] Department of Epidemiology, Rollins School of Public Health, Emory University, Atlanta, GA, USA
[3] Division of Cardiovascular Medicine and the Heart Research Center, Oregon Health & Science University, Portland, OR, USA
[4] Sibley Heart Center Cardiology, Department of Pediatrics, Children's Healthcare of Atlanta, Emory University, Atlanta, GA, USA
[5] Kennedy Krieger Institute, Baltimore, MD, USA
[6] Department of Physiology and the Institute for Genetic Medicine, Johns Hopkins University School of Medicine, Baltimore, MD, USA
[7] Population Health Sciences Institute, Faculty of Medical Sciences, Newcastle University, Newcastle upon Tyne, UK
[8] Division of Cardiovascular Sciences, Faculty of Biology, Medicine and Health, University of Manchester, Manchester, UK
[9] Human Genetics Center; Department of Epidemiology, Human Genetics, and Environmental Sciences, UTHealth School of Public Health, Houston, TX, USA
[10] Division of Cardiology, Children's Hospital of Philadelphia, Philadelphia, PA, USA
[11] Department of Pediatrics, Perelman School of Medicine, University of Pennsylvania, Philadelphia, PA, USA
[12] Department of Surgery, Yale School of Medicine, New Haven, CT, USA
[13] The Ward Family Heart Center, Section of Cardiac Surgery, Children's Mercy Hospital, Kansas City, MO, USA
[14] College of Biosciences, Kansas City University of Medicine and Biosciences, Kansas City, MO, USA
[15] Texas Children's Hospital, Houston, TX, USA

[16] Heart Center, Nationwide Children's Hospital, Columbus, OH, USA
[17] Departments of Pediatrics, Pathology, and Human Genetics, The University of Chicago, Chicago, IL, USA
[18] Department of Pediatrics, Emory University School of Medicine, Atlanta, GA, USA

SUPPLEMENTARY METHODS

Target dataset for primary polygenic risk score analysis

Imputed samples

There originally were 459 samples with Down syndrome (DS), including 211 atrioventricular septal defect (AVSD) cases (which we refer to as DS+AVSD cases) and 248 controls with structurally normal hearts (which we refer to as DS+NH controls), all of whom had available Affymetrix Genome-Wide Human SNP 6.0 array genotype data. These 459 samples included the 210 cases and 242 controls analyzed in the prior genome-wide association study (GWAS) of DS-associated AVSD.[1] Using PLINK1.9 (version 1.90b6.6)[2,3] and R (version 3.4.1),[4] we applied standard GWAS quality control (QC) procedures, excluding subjects for sex discordance, outlier heterozygosity rates (+/- 3 standard deviations [SD] from the mean), missing > 3% of genotypes, and one subject from each pair with proportion of alleles shared identical by descent (IBD) > 0.1875. Variant filters included missing for > 5% of samples, minor allele frequency (MAF) < 0.01, Hardy-Weinberg equilibrium (HWE) mid-p-value < 0.00001 (among controls), and significantly different rates of missingness in cases versus controls (p < 0.00001). We then used principal component analysis (PCA) to identify and remove any population outliers, which involved identifying and removing non-European samples using the HapMap3[5] dataset as a population reference (we identified ancestral outliers based on the Anderson et al. 2010 protocol[6]). All together, these QC steps yielded a dataset with 207 DS+AVSD cases and 234 DS+NH controls, and 612,125 autosomal single nucleotide polymorphisms (SNP), excluding chromosome 21.

For these samples, we then performed genotype imputation using the Michigan Imputation Server.[7] Prior to imputation, all alleles were aligned to the (+) strand, and we used a program[8] written by the McCarthy Group to check our dataset against the Haplotype Reference Consortium (HRC) panel and ensure that our data were properly configured for imputation using the HRC panel. We then submitted the DS dataset to the Michigan Imputation Server, for imputation based on the HRC panel (version r1-1 2016),[9] which includes 32,470 samples predominantly of European ancestry.

The post-imputation files included 38,596,402 autosomal variants (all SNPs). Mean correlation between true and imputed genotypes for the ~600,000 genotyped SNPs was 0.990, suggesting high quality imputation. Considering all post-imputation variants, those with MAF ≥ 0.05 (5,349,403 variants) had mean imputation $r^2 = 0.971$, those with 0.01 ≤ MAF < 0.05 (2,300,344 variants) had mean $r^2 = 0.882$, and those with MAF < 0.01 (30,946,655 variants) had mean $r^2 = 0.180$. This indicates good imputation quality for variants with common or moderate MAF. We decided to drop variants with MAF < 0.01, those missing for more than 2% of samples, those with a maximum imputed genotype probability < 0.80, and those with imputation $r^2 < 0.80$.

We then applied standard GWAS QC to the imputed dataset. We dropped one sample with an outlying heterozygosity rate (> 3 SDs below the mean). No samples were dropped for excess missing genotypes (all had < 1% missingness). Following removal of the single sample, we again excluded variants missing for > 2% of individuals and those with MAF < 0.01, and also dropped variants with HWE mid-p-value < 0.00001 and those with significant differences in missing genotype rate between cases and controls ($p < 0.05$). We also removed variants with A/T, T/A, C/G, and G/C

alleles which can be difficult to match between datasets due to strand ambiguity. This left a dataset with 440 samples (206 DS+AVSD cases, 234 DS+NH controls) and 5,079,537 autosomal SNPs.

Whole genome sequencing samples

Starting from the previously described post-QC whole genome sequencing (WGS) dataset, which included 175 samples (148 DS+AVSD cases, 27 DS+NH controls), we applied additional variant filters in order to more closely match the variant QC procedures which had been applied to the imputed dataset. We removed variants with MAF < 0.01, those missing for > 2% of samples, and indels, leaving a WGS dataset with 175 samples and 4,173,676 autosomal SNPs (excluding chromosome 21).

Merging WGS and imputed samples

Coordinates for the WGS dataset were based on human genome build 38 (hg38), while those for the imputed dataset were based on human genome build 19 (hg19). Prior to merging the datasets, we used the University of California Santa Cruz (UCSC) Genome Browser[10] LiftOver tool to convert the WGS data coordinates from hg38 to hg19, and also modified dbSNP Reference numbers (rsIDs) for each variant as needed using an external file based on HRC panel variants containing hg19 rsIDs and coordinates. We chose to convert the WGS data to hg19 rather than converting the imputed data to hg38 as a matter of convenience, given the polygenic risk score (PRS) training files we used had hg19 coordinates.

As one additional step prior to merging the WGS and imputed datasets, we compared allele frequencies for SNPs in each dataset in order to identify any instances where allele frequency for a SNP in one dataset differed considerably from its allele frequency in the other dataset, which could indicate genotyping error for the variant. We identified and removed 77 SNPs with allele frequencies that differed by at least 0.20 between the WGS and imputed datasets.

We then merged the WGS and imputed datasets on rsID, position, and alleles (using PLINK1.9), yielding a single dataset with 615 samples and 2,366,788 SNPs. For all 615 samples missingness was < 1%. An IBD check identified 90 sample duplicates and 1 sample pair with a sibling or child/parent relation. Each of these related pairs involved a WGS sample and an imputed sample (i.e., the duplicates were the result of each sample being represented in both the imputed and WGS datasets). For these samples, we kept the data from the WGS dataset as it appeared to be of slightly better quality overall, and we dropped the imputed duplicates. No additional variant QC filters were needed -- all SNPs had missingness ≤ 2% among all samples and ≤ 3% among both cases and controls, all had MAF approximately ≥ 1% (we applied stricter MAF filters during PRS construction), and no SNPs required dropping for HWE violation. Thus, this intermediate data set included 524 samples (263 cases, 261 controls) and 2,366,788 autosomal SNPs.

We next performed PCA, first anchoring our dataset in the HapMap3 dataset and constructing principal components (PC) to identify and remove DS samples with PC values outside of the HapMap3 CEPH/Utah (CEU) cluster (in order to match the European ancestry of the discovery datasets), and then removing the HapMap samples

and performing further outlier removal based only on the DS samples. We constructed PCs for just the DS samples, and removed samples with values > 3 SD from the mean for PC1 or PC2 (which explained most of the genetic variation in the sample). We then reconstructed PCs for the remaining samples and again identified 3 SD outliers for removal, repeating this PCA process until all substantial outliers had been identified and removed. This PCA approach identified 37 sample outliers for removal.

As a final step in preparing the DS target dataset for PRS analysis, we removed the extended major histocompatibility complex region (chromosome 6, ~25000000-34000000, human genome build 19), which is a region of extended high linkage disequilibrium that can overly influence PRS results. Our final data set included 487 samples (245 DS+AVSD cases, 242 DS+NH controls) and 2,351,951 autosomal SNPs (excluding chromosome 21). The multiple steps involved in generating this final data set for the primary PRS analyses are presented as a flowchart in Supplementary Fig. S1.

Target dataset for secondary PRS analysis

Our secondary PRS analyses examined the contribution by alleles on the trisomic chromosome 21 to a polygenic component for DS-associated AVSD. To do this, we compared PRS results based on polygenic scores generated using all autosomes (including chromosome 21) to PRS results based on scores using all autosomes except for chromosome 21.

We analyzed the same set of target samples as for the primary analyses (245 DS+AVSD cases, 242 DS+NH controls), 158 of whom had WGS data for chromosome 21, and 329 of whom had Affymetrix Genome-Wide Human SNP 6.0 array genotype

data for chromosome 21 (given the complexities of imputing trisomic genotypes, we did not have imputed data for these 329 samples). Given that trisomic data cannot be represented by the PLINK1.9 binary format, we handled these chromosome 21 data separately from the other chromosomes. Prior to merging chromosome 21 data for these WGS and array samples, we applied certain QC filters. None of the 158 WGS samples nor the 329 array samples had an excess of missing genotypes for chromosome 21 (all had approximately 5% or less missingness). For variant QC, we excluded SNPs missing for > 5% of samples, as well as SNPs with A/T, T/A, C/G, and G/C alleles which can be difficult to match between datasets due to strand ambiguity. We also removed SNPs with substantially different allele frequencies between the WGS and array datasets (we determined that a frequency difference of ≥ 0.125 was an appropriate threshold for these chromosome 21 datasets). Post-merger, we removed SNPs with excess missingness specifically among cases or controls (missing for > 3% of cases or > 3% of controls), and we also excluded SNPs that were monoallelic in the full sample. These steps yielded a merged chromosome 21 dataset with 487 samples and 3,984 SNPs.

We then took the dataset used for the primary analyses (487 samples and 2,351,951 autosomal SNPs, excluding chromosome 21), and limited it to SNPs on the Affymetrix Genome-Wide Human SNP 6.0 array, leaving 389,544 SNPs. This was done since the chromosome 21 data were also necessarily limited to the array SNPs. We used these SNP-array-level genotype data, both with and without the chromosome 21 data, in order to perform the secondary PRS analyses.

SUPPLEMENTARY TABLES

Supplementary Table S1: PRS results using discovery GWAS of 2,594 mixed CHD cases and 5,159 controls and SNPs with MAF ≥ 0.35. 'Threshold' indicates that SNPs with discovery GWAS p-values below the threshold were used for PRS construction, and 'No. SNP' is the corresponding number of SNPs used for scoring. OR: Odds ratio per standard deviation increase in PRS, CI: Confidence interval (corresponding to uncorrected p-value), Nag. r^2: Nagelkerke's r^2, P_{unadj}: Uncorrected p-value, P_{adj}: P-value corrected for multiple correlated tests.

Threshold	No. SNP	OR	95% CI	Nag. r^2	P_{unadj}	P_{adj}
**1e-05**	1	1.12	0.91-1.38	0.24%	0.278	> 0.15
**1e-04**	5	1.19	0.96-1.47	0.54%	0.107	> 0.15
**0.001**	93	1.27	1.03-1.57	1.03%	0.027	> 0.15
**0.005**	328	1.25	1.01-1.54	0.91%	0.037	> 0.15
**0.01**	597	1.35	1.09-1.67	1.61%	0.006	> 0.15
**0.05**	2,421	1.25	1.02-1.54	0.95%	0.033	> 0.15
**0.1**	4,275	1.28	1.03-1.57	1.09%	0.023	> 0.15
**0.2**	7,590	1.22	0.99-1.50	0.75%	0.059	> 0.15
**0.3**	10,432	1.18	0.96-1.46	0.54%	0.108	> 0.15
**0.4**	12,982	1.11	0.91-1.37	0.22%	0.303	> 0.15
**0.5**	15,197	1.12	0.91-1.38	0.25%	0.278	> 0.15
**1**	22,507	1.09	0.89-1.34	0.15%	0.389	> 0.15

SUPPLEMENTARY FIGURES

Supplementary Figure S1: Flowchart showing the multiple steps involved in generating the final data set for the primary PRS analyses.

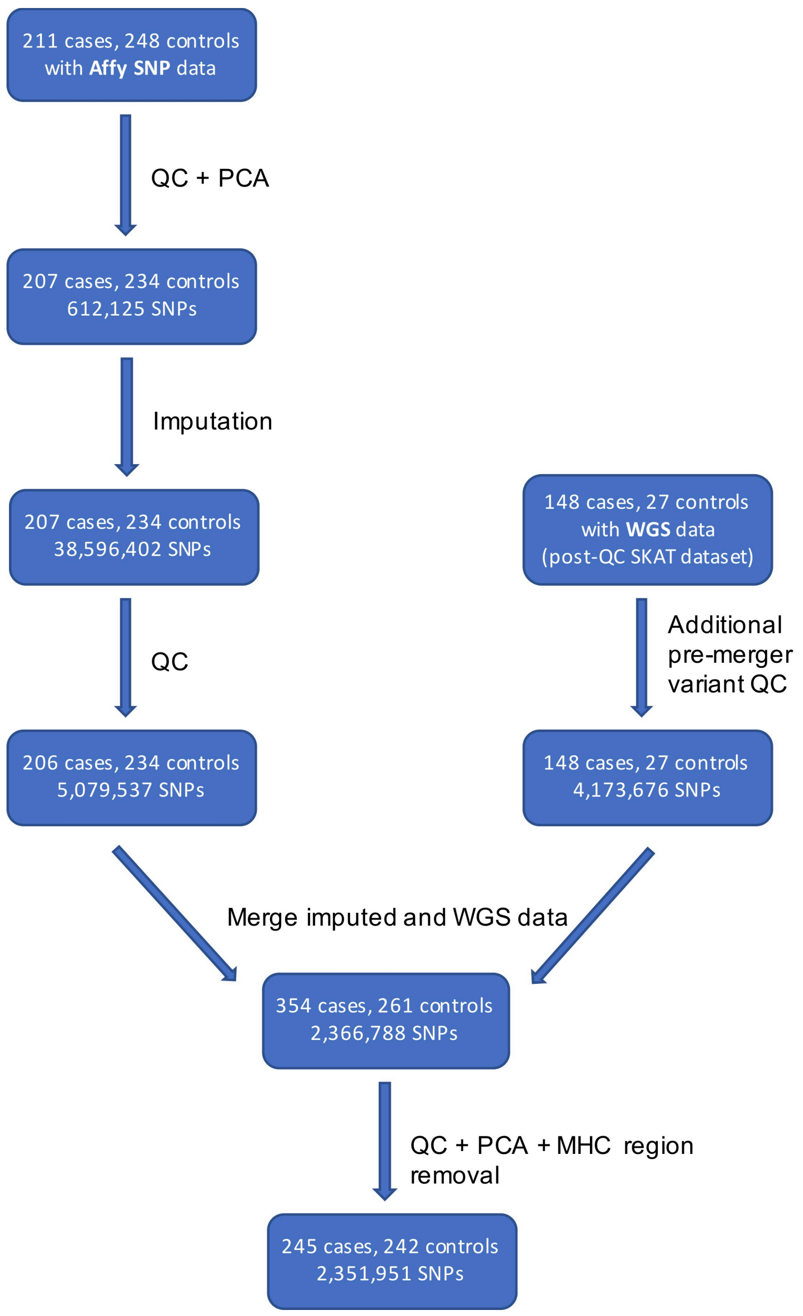

Supplementary Figure S2. Representative SKAT-O Manhattan plot and QQ plot of common variants, generated from analyses using the whole exome sequencing dataset. Each dot represents a gene in the SKAT-O analysis, ordered by chromosome. No gene reached Bonferroni significance (red horizontal line), however 30 genes showed a nominal significance level of p < 0.001 (blue horizontal line).

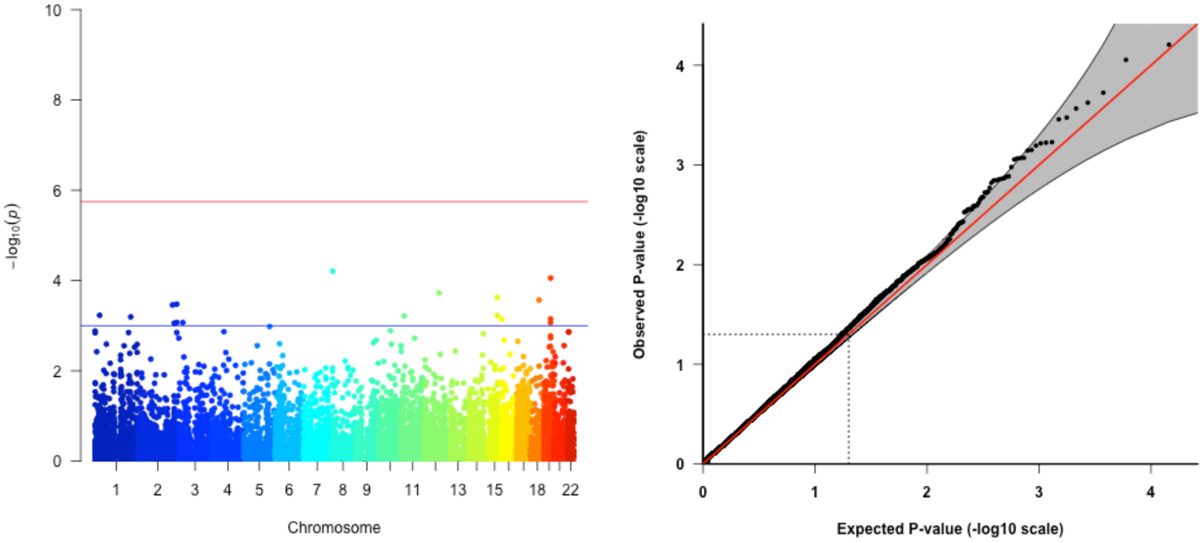

Supplementary Figure S3: PRS results using discovery GWAS of 2,594 mixed CHD cases and 5,159 controls and various MAF thresholds. MAF thresholds were applied to the discovery GWAS; SNPs with MAF below the threshold were excluded from PRS construction. Top row: Each plot displays odds ratio per standard deviation in PRS and the corresponding 95% confidence interval (y-axis) for PRS constructed based on particular discovery GWAS p-value thresholds (x-axis). P_{adj} are adjusted p-values (corrected for multiple correlated tests). 95% CIs correspond to unadjusted p-values. Bottom row: Each plot displays Nagelkerke's r^2 (y-axis) for PRS constructed based on particular discovery GWAS p-value thresholds (x-axis). Numbers above each r^2 bar are the number of SNPs used to construct PRS at that particular p-value threshold and MAF filter combination.

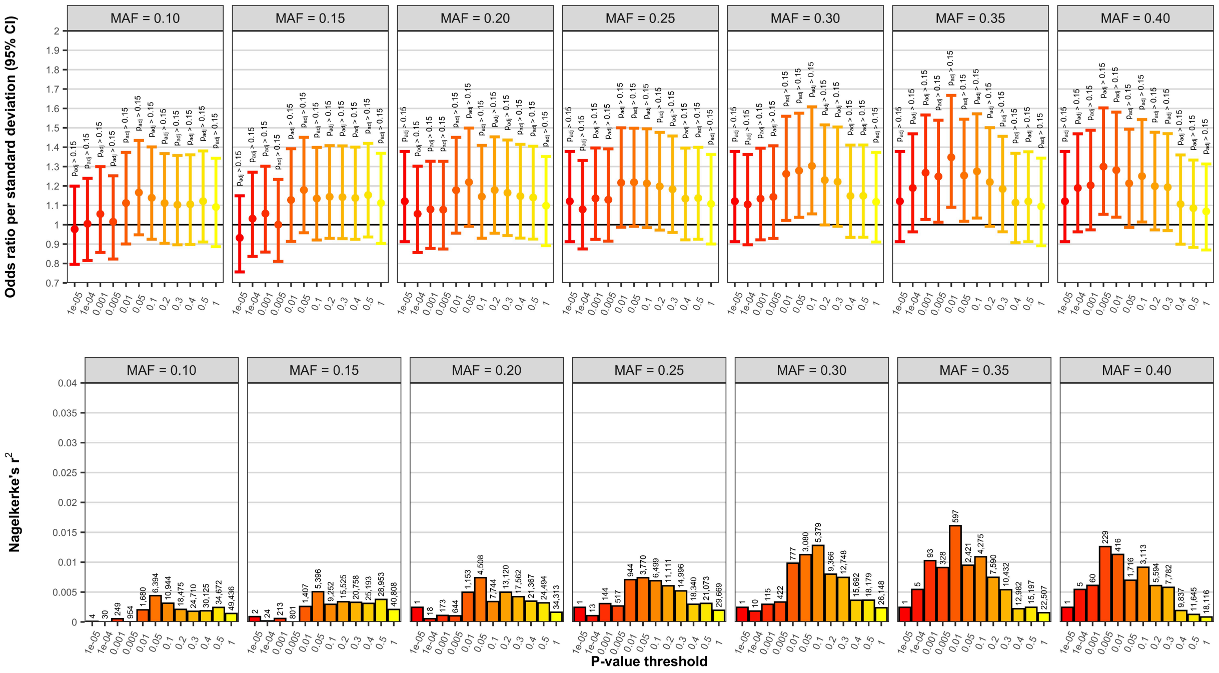

Supplementary Figure S4: PRS results using discovery GWAS of 406 mixed CHD cases and 2,976 controls and various MAF thresholds. MAF thresholds were applied to the discovery GWAS; SNPs with MAF below the threshold were excluded from PRS construction. Top row: Each plot displays odds ratio per standard deviation in PRS and the corresponding 95% confidence interval (y-axis) for PRS constructed based on particular discovery GWAS p-value thresholds (x-axis). P_{adj} are adjusted p-values (corrected for multiple correlated tests). 95% CIs correspond to unadjusted p-values. Bottom row: Each plot displays Nagelkerke's r^2 (y-axis) for PRS constructed based on particular discovery GWAS p-value thresholds (x-axis). Numbers above each r^2 bar are the number of SNPs used to construct PRS at that particular p-value threshold and MAF filter combination.

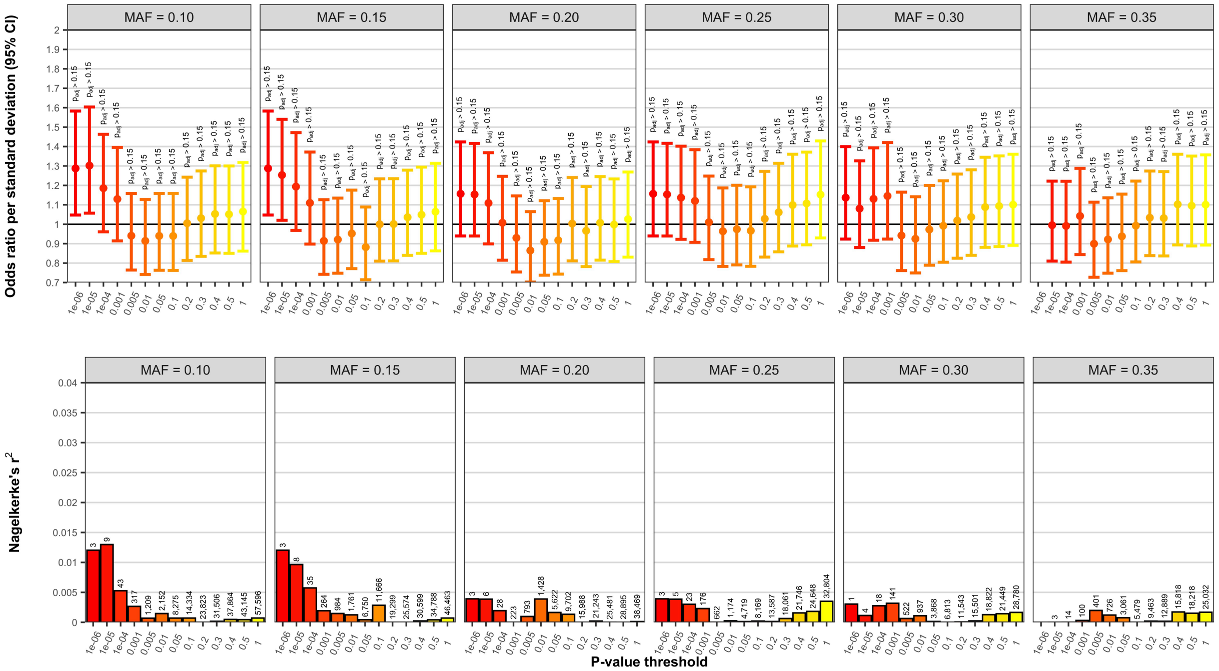

Supplementary Figure S5: PRS results using meta-analysis of two GWAS as discovery dataset and employing inverse-variance-weighted SNP effects for scoring, for various MAF thresholds. MAF thresholds were applied to the discovery GWAS; SNPs with MAF below the threshold were excluded from PRS construction. Top row: Each plot displays odds ratio per standard deviation in PRS and the corresponding 95% confidence interval (y-axis) for PRS constructed based on particular discovery GWAS p-value thresholds (x-axis). P_{adj} are adjusted p-values (corrected for multiple correlated tests). 95% CIs correspond to unadjusted p-values. Bottom row: Each plot displays Nagelkerke's r^2 (y-axis) for PRS constructed based on particular discovery GWAS p-value thresholds (x-axis). Numbers above each r^2 bar are the number of SNPs used to construct PRS at that particular p-value threshold and MAF filter combination.

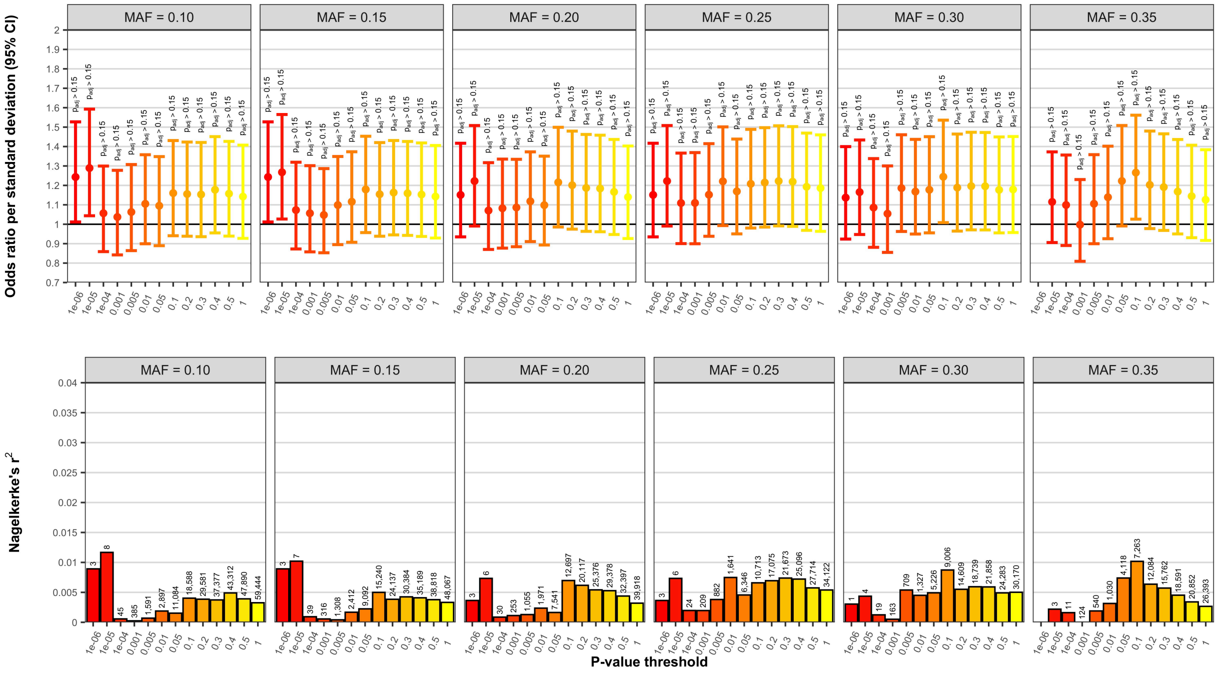

Supplementary Figure S6: PRS results for all autosomes *excluding* chromosome 21. These analyses used the discovery GWAS of 2,594 mixed CHD cases and 5,159 controls. Various MAF thresholds were applied to the discovery GWAS; SNPs with MAF below the threshold were excluded from PRS construction. Top row: Each plot displays odds ratio per standard deviation in PRS and the corresponding 95% confidence interval (y-axis) for PRS constructed based on particular discovery GWAS p-value thresholds (x-axis). P_{adj} are adjusted p-values (corrected for multiple correlated tests). 95% CIs correspond to unadjusted p-values. Bottom row: Each plot displays Nagelkerke's r^2 (y-axis) for PRS constructed based on particular discovery GWAS p-value thresholds (x-axis). Numbers above each r^2 bar are the number of SNPs used to construct PRS at that particular p-value threshold and MAF filter combination.

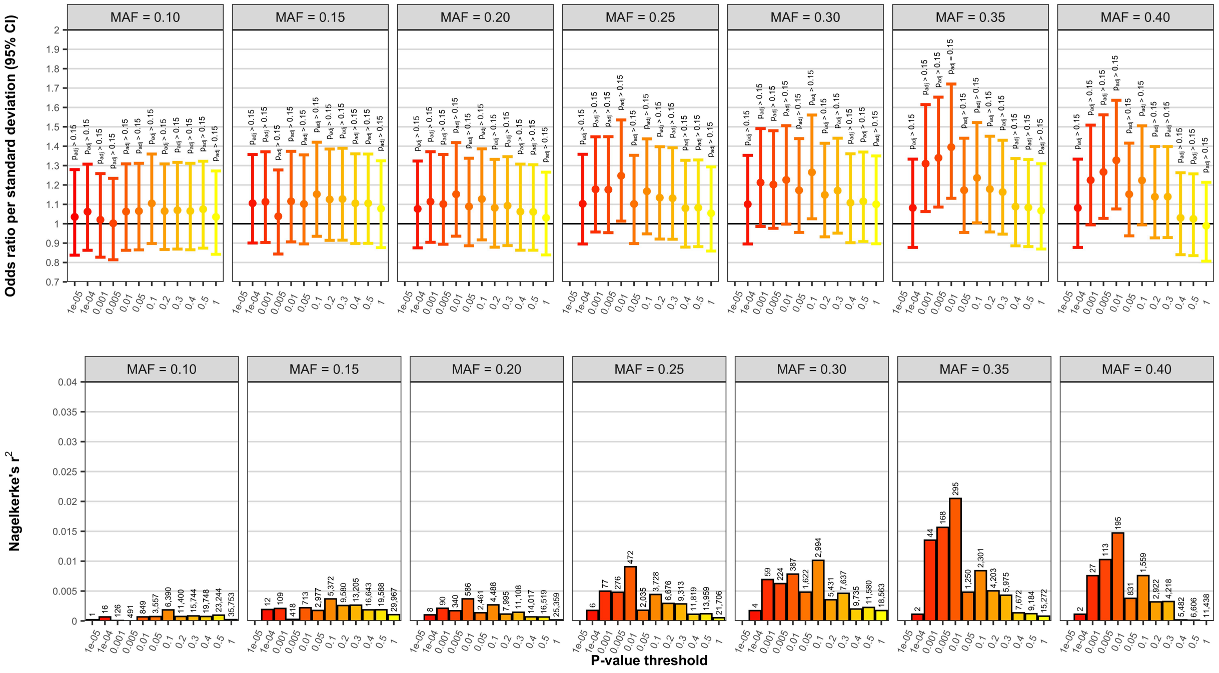

Supplementary Figure S7: PRS results for all autosomes *including* chromosome 21. These analyses used the discovery GWAS of 2,594 mixed CHD cases and 5,159 controls. Various MAF thresholds were applied to the discovery GWAS; SNPs with MAF below the threshold were excluded from PRS construction. Top row: Each plot displays odds ratio per standard deviation in PRS and the corresponding 95% confidence interval (y-axis) for PRS constructed based on particular discovery GWAS p-value thresholds (x-axis). P_{adj} are adjusted p-values (corrected for multiple correlated tests). 95% CIs correspond to unadjusted p-values. Bottom row: Each plot displays Nagelkerke's r^2 (y-axis) for PRS constructed based on particular discovery GWAS p-value thresholds (x-axis). Numbers above each r^2 bar are the number of SNPs used to construct PRS at that particular p-value threshold and MAF filter combination.

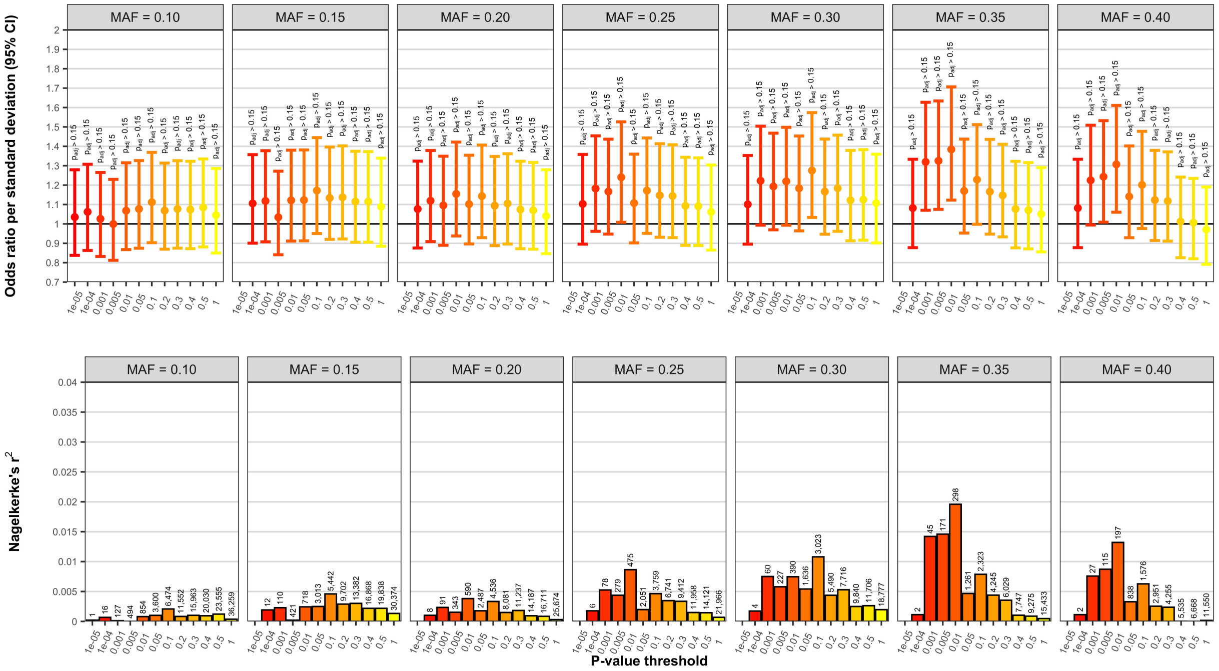

Supplementary Figure S8: Maximum variance in target phenotype that can be explained by PRS (y-axis: liability scale r^2) given a range of training sample sizes (x-axis: number of cases in thousands). Assumptions: training sample with case:control ratio of 1:2 (same as ratio for larger of the two independent CHD discovery datasets); target sample with case:control ratio of 1:1 (same as ratio for DS target dataset); prevalence of CHD in training population is 1%; prevalence of AVSD in DS target population is 20%; 100,000 independent variants in the training SNP panel; genetic effects for training and target samples are identical (correlation = 1); proportion of SNPs in the training set panel that affect the training phenotype is 1%, 10% or 100%. For plot **A**, amount of variance in the training phenotype explained by the training set SNP panel (Vg_{train}) is 15%; for plot **B** Vg_{train} is 25%; for plot **C** Vg_{train} is 35%. Solid black horizontal line marks the maximum r^2 that can be explained by PRS using an infinitely large training sample size (given the assumed parameters). Vertical orange line marks the number of CHD cases in the larger of the two independent discovery datasets (2,594 cases).

A.

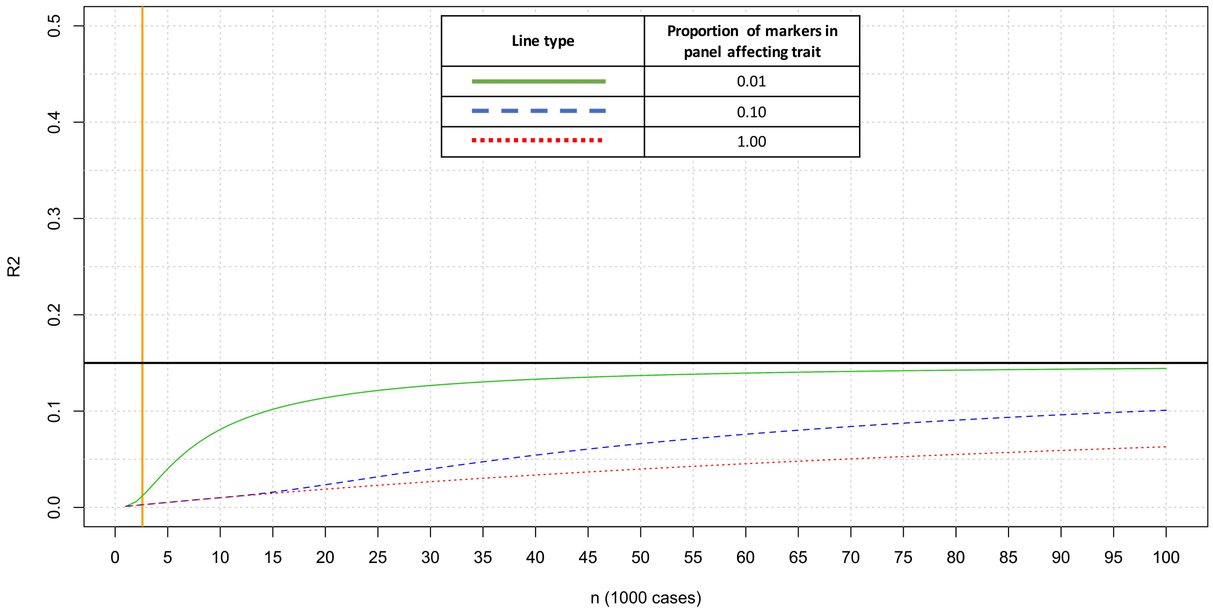

B.

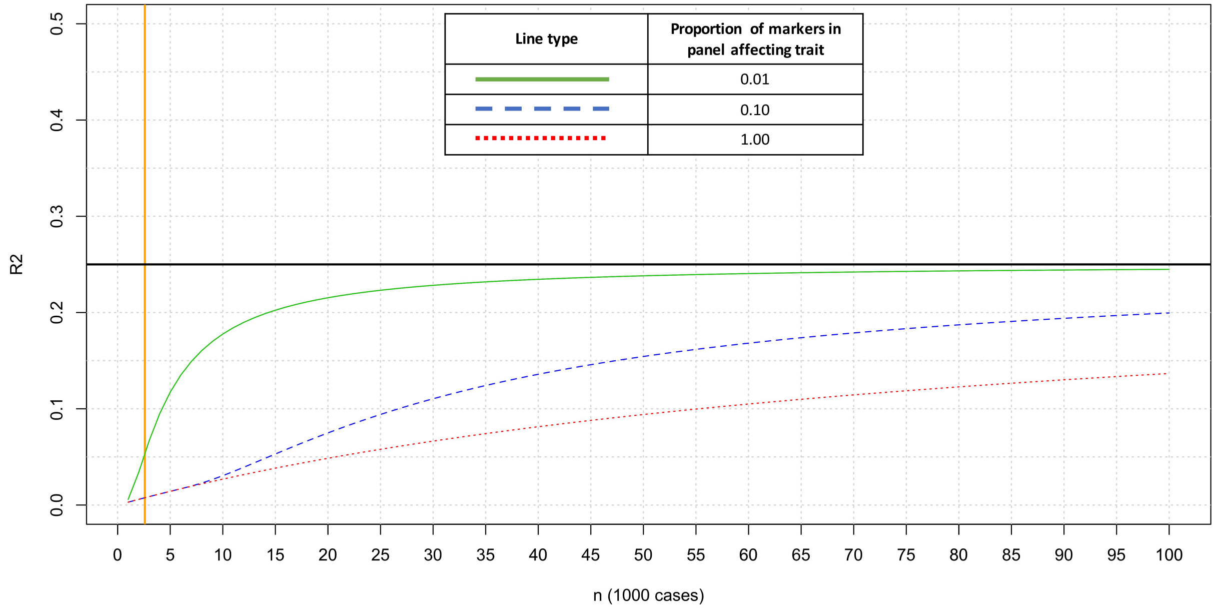

Line type
Proportion of markers in panel affecting trait
0.01
0.10
1.00
R2
0.0
0.1
0.2
0.3
0.4
0.5
0 5 10 15 20 25 30 35 40 45 50 55 60 65 70 75 80 85 90 95 100
n (1000 cases)

C.

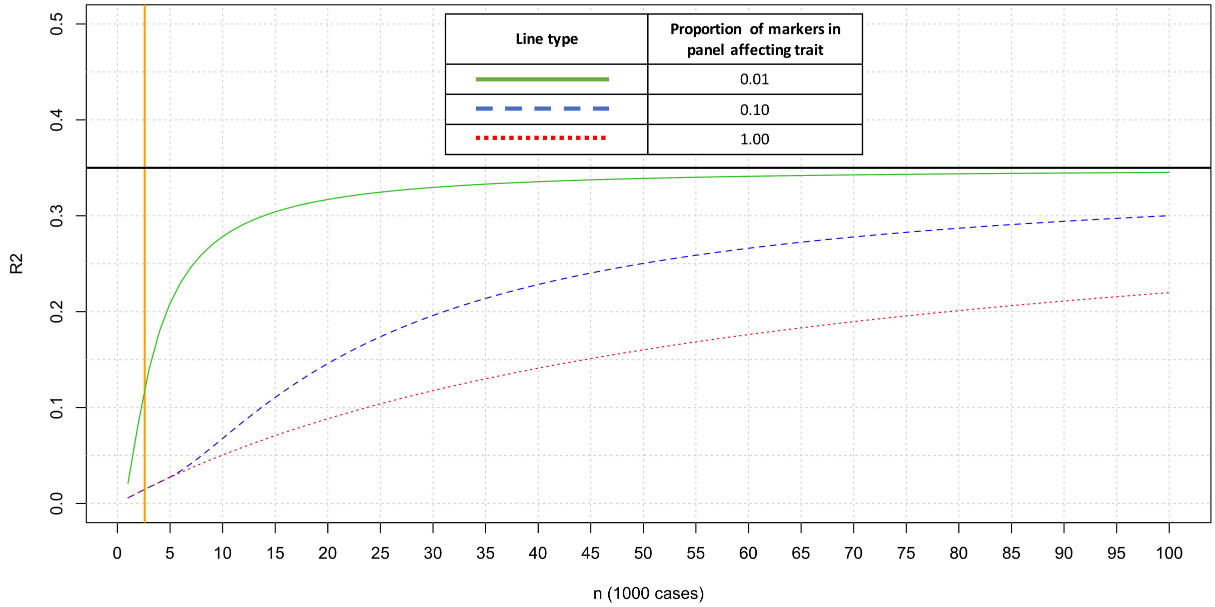

Line type
Proportion of markers in panel affecting trait
0.01
0.10
1.00
R2
0.0
0.1
0.2
0.3
0.4
0.5
0 5 10 15 20 25 30 35 40 45 50 55 60 65 70 75 80 85 90 95 100
n (1000 cases)

REFERENCES

1. Ramachandran, D. *et al.* Genome-Wide Association Study of Down Syndrome-Associated Atrioventricular Septal Defects. *G3* **5**, 1961–1971 (2015).
2. Chang, C. C. *et al.* Second-generation PLINK: rising to the challenge of larger and richer datasets. *GigaScience* **4**, 7 (2015).
3. Purcell, S. M. & Chang, C. C. PLINK [v1.9b6.6]. www.cog-genomics.org/plink/1.9/.
4. R Core Team. R: A language and environment for statistical computing. https://www.R-project.org/ (R Foundation for Statistical Computing, 2017).
5. International HapMap Consortium. A haplotype map of the human genome. *Nature* **437**, 1299–1320 (2005).
6. Anderson, C. A. *et al.* Data quality control in genetic case-control association studies. *Nat. Protoc.* **5**, 1564–1573 (2010).
7. Das, S. *et al.* Next-generation genotype imputation service and methods. *Nat. Genet.* **48**, 1284–1287 (2016).
8. Rayner, W. Script to check plink .bim files against HRC/1000G for strand, id names, positions, alleles, ref/alt assignment [v 4.2.9]. https://www.well.ox.ac.uk/~wrayner/tools/.
9. McCarthy, S. *et al.* A reference panel of 64,976 haplotypes for genotype imputation. *Nat. Genet.* **48**, 1279–1283 (2016).
10. Kent, W. J. *et al.* The human genome browser at UCSC. *Genome Res.* **12**, 996–1006 (2002).
